# Effect of early experience on neuronal and behavioral responses to con- and heterospecific odors in closely related Mus taxa: epigenetic contribution in formation of precopulatory isolation

**DOI:** 10.1186/s12862-019-1373-8

**Published:** 2019-02-26

**Authors:** Elena Kotenkova, Alex Romachenko, Alexander Ambaryan, Aleksei Maltsev

**Affiliations:** 1 Severtsov Institute of Ecology and Evolution RAS, Leninsky Prospect, 33, 119071 Moscow, Russia; 2 Institute of Cytology and Genetics SB RAS, Prospekt Lavrentyeva 10, 630090 Novosibirsk, Russia

**Keywords:** *Mus musculus*, *Mus spicilegus*, Cross-fostering, Early olfactory experience, Main olfactory bulb, Accessory olfactory bulb, Learning, MEMRI

## Abstract

**Background:**

The most effective learning occurs during sensitive periods. Olfactory plasticity to main social olfactory cues is limited to a critical period to a large degree. The objective was to evaluate the influence of early olfactory experience on the behavioral and neuronal responses of males to con- and heterospecific odors of receptive females in two species, *M. musculus* (subspecies *musculus*, *wagneri*) and *M. spicilegus*, and thus to determine the potential role of epigenetic contribution in the formation of precopulatory isolation.

**Results:**

Males were reciprocally cross-fostered shortly after the birth and were tested for response to con- and heterospecific urine odors of estrus females using two-choice tests at 70–85 days of age. Neuronal activity of non- and cross-fostered males was evaluated at 90–110 days of age in the MOB and AOB to con- and heterospecific female odor using fMRI (MEMRI). Non-cross-fostered males of three taxa demonstrated a strong preference for odor of conspecific females compared to odor of heterospecific ones. *Spicilegus*-nursed *musculus* preferred odor of heterospecific females. *Wagneri*-nursed *spicilegus* and *spicilegus*-nursed *wagneri* did not demonstrate significant choice of con - or heterospecific female odor. The level of MRI signal obtained from the evaluation of manganese accumulation in AOB neurons was significantly higher when the odor of conspecific estrus females was exposed, compared to urine exposure of heterospecific females. The response pattern changed to the opposite in males raised by heterospecific females. Response patterns of neuronal activity in the MOB to con- and heterospecific female odors were different in cross-fostered and control males.

**Conclusion:**

The maternal environment, including odor, had a greater effect on the level of MRI signal in the AOB than the genetic relationships of the recipient and the donor of the odor stimulus. Behavioral and neuronal responses to con- and heterospecific odors changed in closely related Mus taxa as a result of early experience. We demonstrated the importance of early learning in mate choice in adulthood in mice and the possibility of epigenetic contribution in the formation of precopulatory reproductive isolation.

## Background

Learning occurs in most animal taxa and in different environmental contexts. It is widespread in nature and, thus, might be important in evolutionary processes [1, 2]. The classic demonstrations of how learning can affect mating preferences and display traits come from field and laboratory studies of sexual imprinting and song learning in birds [3–5]. Сonspecific mate preference and assortative mating can often result from imprinting on related individuals [6–8]. Sexual imprinting establishes a “sort of consciousness of the species in the young bird” [9] which is then used in mate choice. The individual learned phenotypic traits of parents and/or siblings, such as visual, auditory or olfactory ones, result in the learner being able to discriminate its own species and sex of conspecifics [10–12]. The results of many of field and laboratory investigations were reviewed and discussed, confirming the essential importance of different forms of learning in the evolutionary process. Learned mate preferences and learned display traits can contribute to sexual selection, the evolution of reproductive isolation, population divergence, and sexual conflict [1, 10, 13–15]. It should be noted that the main model groups of these studies were some species of fish and birds, in which visual and auditory signals have the leading role in communication [1, 5–7, 11, 16]. The significance of olfactory cues has been studied much less [12, 17–19].

The most effective learning occurs during sensitive periods. Sensitive periods are viewed as times during development when experience exerts a very strong influence on the brain and on behavior. Critical periods are a special class of sensitive periods that result in irreversible changes or greatly modifications in brain function, but the possibility of their partial recovery under certain environmental conditions is preserved. During sensitive periods, experience is thought to instruct neural circuits to process or represent information in ways that are adaptive for the individual [20]. Axon elaboration and synapse formation, as well as axon and synapse elimination are two mechanisms that have been shown to alter circuit architecture in visual and auditory systems during sensitive periods. Synapse consolidation is a third mechanism that could underlie fundamental architectural changes that result from experience during sensitive periods [20]. These mechanisms could account for the persistence of learning that occurs during sensitive periods. When a circuit can select from a large range of potential patterns of connectivity, the effect of experience can have an enormous impact on circuit connectivity. These changes are possible as a result of neuroplasticity. The most recent definition of neuroplasticity is based on the permanent changes in the properties of nerve cells that occur under the influence of environmental stimuli or from a break in the continuity or other damage to the nervous system [21]. Mechanisms of neural plasticity of visual, auditory and olfactory systems during sensitive periods are considered in reviews [20, 22–25].

Compared to other sensory systems, the olfactory system has a high plasticity not only during early period of ontogenesis, but also during adulthood of animals. This phenomenon is based on the processes of continuous regeneration of the olfactory epithelium and the epithelium of the vomeronasal organ. Olfactory bulbs (OB) retain the ability to neurogenesis throughout the life cycle of the animal [26, 27]. Olfactory and vomeronasal epithelium undergo continuous cell turnover, and newly generated interneurons arising from the subventricular zone of telencephalon are added to main (MOB) and accessory olfactory bulbs (AOB) [28]. Three forms of intraspecies olfactory learning (mate recognition in mice, maternal recognition of offspring in sheep and early olfactory learning in rats and rabbits) are studied relatively well. It was demonstrated that synaptic plasticity underlying these forms of olfactory learning may occur in the glomeruli of the OB at the first level of olfactory processing and inhibitory interneurons play a critical role in olfactory learning [24].

Nevertheless, olfactory plasticity to main social olfactory cues is limited to a critical period to a large degree. In this period exposure to the odor might change responses to con- and heterospecific odors in some species of mammals, but not in other species. If the range of potential patterns of responses is highly constrained by genetic programs, the effect of experience in critical periods is correspondingly small, see reviews [19, 29]. Of particular interest is the question of how epigenetic effects, such as imprinting and other forms of learning during early experience can influence on mate choice and preference of adult individuals. Sexually mature rodents typically display strong behavioral preferences for conspecific odors from opposite-sex individuals compared to odors of heterospecific ones [30–33].

In most mammals, a major component of social environment is provided by mothers [34], and of particular interest is to what extent the epigenetic effects can depend on the maternally provided environment [35]. The approach of cross-fostering of offspring to lactating females of different species has been used for research on the role of early olfactory experience in adult odor preferences [12, 17, 18, 36, 37]. In mammals, mothers nurse and care for the pups and the mother’s odor can induce a positive conditioned reflex. It is difficult to separate imprinting from other forms of learning in the formation of various behavioral reactions. Therefore, we will use the term “early olfactory experience”.

A shift in preference toward odor of the foster parent indicates that species-specific odors are learned via the early experience with the foster parent. The observed changes are characterized by increased attraction to the heterospecific foster species rather than a complete reversal of species preference [38, 39] or decreased attraction to the conspecific species [17, 40, 41]. The olfactory system and olfactory behavior thus provides an attractive model to investigate processes involving interplay between genetic and epigenetic influences and their role in evolutionary process, especially development of precopulatory reproductive isolation.

Data from studies that addressed the effect of early olfactory experience on the subsequent response to conspecific odor in house mice (different strains of laboratory mice and wildliving *Mus domesticus*) are contradictory. Some authors reported that adult male and female mice, fostered by parents of the genera *Baiomys*, *Peromyscus* or Norway rat *Rattus norvegicus*, investigated longer the odor of foster species or preferentially stayed in a compartment of the chamber with this odor as compared to conspecific odor [17, 42]. According to the results of Wuensch [18], the early olfactory experience did not affect the reaction to conspecific odor in house mice, but had a considerable effect on the response to the odor of fostered species (rat). The results of Kirchhof-Glazier [43] did not agree with those described above, since the author did not detect any influence of early olfactory experience on the behavioral or physiological reactions in laboratory mouse females (strain CjL/C) fostered by deer mice *Peromyscus maniculatus* from the first day of life. Contradictions can be explained by differences in methods and genetic peculiarities of experimental mice and fostered species.

Individuals of closely related allopatric, parapatric, and sympatric taxa of species group *Mus musculus* sensu lato discriminate odors of their own species and heterospecifics and usually prefer odor of conspecifics [44–48]. Controversy persists over the taxonoimic status of the two commensal taxa, *Mus (musculus) musculus* Linnaeus, 1758 [49], and *Mus (musculus) domesticus* Schwarz & Schwarz, 1943 [50, 51], but for simplicity (and following Sage et al. [52]) throughout this paper we consider these two taxa as distinct species. The species *Mus musculus* (subspecies *M. m. musculus*) and *M. spicilegus* are sympatric, *M. m. wagneri* and *M. spicilegus* are allopatric [52–54]. Individuals of *M. musculus* and *M. spicilegus* that we selected for testing investigated conspecific urine odor significantly longer than heterospecific urine odor (including the odor from closely related species) in different two-choice combinations, regardless of the sex of the odor donors [44, 47, 55]. According to our preliminary data early olfactory experience to alter the response of *M. musculus* and mound-building mice *M. spicilegus* to con- and heterospecific odors [37]. Here we used the same standardized two-choice odor test in studies performed over many years, and this permitted comparison of results obtained at different times [47].

Previously we showed that exposure of *M. domestius* males to conspecific receptive female bedding induced Fos-immunoreactivity in both apical and basal zones of vomeronasal organ (VNO), which suggests the multicompound nature of the chemical signal. Fos-positive cells were located mainly in the rostral part of VNO [56]. In the response to exposure of receptive *M. spicilegus* female bedding to conspecific male we observed Fos-immunoreactivity in receptor VNO epithelium mainly in basal zone. Thus, the pattern of VNO receptor cells activation in response to stimulation with receptive female odor was different in males of the two species. The specific pattern of the activation in the sensory epithelium was absent when we exposed males to heterospecific female bedding. For the males of three taxa, *M. musculus*, *M. spicilegus*, *M. domesticus*, in response to stimulation with conspecific receptive female bedding we observed a clear pattern of activation in the caudal part of the AOB which receives projections from the basal VNO zone where receptors binding to higher molecular weight substances are expressed [57, 58]. At the same time in response to exposure of receptive *M. spicilegus* female bedding to *M. musculus* and *M. domesticus* males, we did not observe any Fos-immunoreactivity in caudal zone of AOB. Heterospecific female odor did not induce neural activation neither at the level of receptor tissue nor at the projecting area of AOB [56]. Taking into account the essential difference in chemical composition of urine *M. domesticus* and *M. spicilegus* [59] these data confirm the point of view that the systems of olfactory communication of sympatric species *M. musculus* and *M. spicilegus* are very different.

*M. musculus* (subspecies *M. m. musculus*) and *M. spicilegus* do not hybridize under natural conditions. Their precopulatory reproductive isolation is provided by multiple mechanisms at different levels of organization: from differences in behavioral patterns of sexual behavior [60] to differences in response to con- and heterospecific olfactory cues [44, 56]. Precopulatory isolating mechanisms can function at the receptor level as well as through different behavioral responses of individuals to olfactory cues upon interactions of potential sexual partners, taken together these provide reliable reproductive isolation for sympatric species under natural conditions [61].

Advances in the study of neural plasticity during the sensitive period of early ontogenesis could be utilized as a model for hypothesizing about the genetic and epigenetic constituents in development of precopulatory reproductive isolation in evolution. We alter maternal environment by cross-fostering such that cross-fostered pups are reared by heterospecific female. The objective of our research was to evaluate the influence of early olfactory learning on the neuronal and behavioral response of males to con- and heterospecific odors of receptive females in two species *M. musculus* and *M. spicilegus* and thus, to determine the potential role of epigenetic contribution in process of formation of precopulatory olfactory isolating mechanisms.

We used one of functional magnetic resonance imaging (fMRI), manganese-enhanced MRI (MEMRI) for investigation of activity of olfactory neurons in males in response to the exposure of the odor of receptive female urine. MEMRI is a comparatively new noninvasive method to map neuronal function and connections [62, 63]. Manganese ion (Mn^2+^) enters neurons through voltage-gated calcium channels [64, 65], can be transported along axons and can cross synapses [66–69]. Mn^2+^ transport across a synapse relies on presynaptic release and postsynaptic uptake, therefore, the amount of Mn^2+^ transported may change depending on the strength of connections if there is plasticity in a neural system [70, 71]. Mn^2+^ can be transported from the nose of a rodent to the OB and the tracing to the OB could be modulated by odorants [72], and MEMRI can be used to map neuronal function and connections in olfactory system [73]. Mn^2+^ is paramagnetic. Paramagnetic ions cause the spin-lattice relaxation time of H_2_O to shorten. The result is that wherever there is accumulation of Mn^2+^ within a tissue, there will be positive contrast enhancement in T1-weighted MRI images [74–78]. That is, the signal intensity of such areas of accumulated Mn^2+^ will appear bright in T1-weighted MRI.

## Methods

### Animals and cross-fostering procedure

Our study was performed with adult male offspring of three taxa of *Mus musculus* s.l. species group reared either by their biological mothers, or by heterospecific foster-mothers (Table. 1).

**Table 1:** Experimental males and summary of treatment conditions

Species of males	Reared by	Fostering or non- fostering group at adulthood	Sample size (number of males)		Age of pup cross-fostering
			Two-choice test	MEMRI	
*spicilegus*	*spicilegus*	*spicilegus*	7	14	–
*spicilegus*	*wagneri*	*spicilegus*_w_	10	10	48–60 h
*spicilegus*	*musculus*	*spicilegus*_mus_	4	2	120–132 h
*wagneri*	*wagneri*	*wagneri*	9	11	–
*wagneri*	*spicilegus*	*wagneri*_sp_	6	10	48–60 h
*musculus*	*musculus*	*musculus*	12	14	–

*w wagneri*, *sp spicilegus*, *mus musculus*

The parental mice used in this study were laboratory-born, 3–4 months old (F_3–4_ generation) descendants of *M. m. musculus* trapped in Moscow and the Moscow region, *M. m. wagneri* trapped in the Astrakhan region, and *M. spicilegus* collected in the Rostov region. All subjects were bred in our laboratory in A. N. Severtsov Institute of Ecology and Evolution RAS.

All mice were housed in Macrolon cages type III (265 × 180 × 420 mm, ZOONLAB GmbH, Germany), which contained sawdust, food (Special mixed fodder for mice, Russia) and water ad libitum. Housing of the animals was standardized by 14:10 h light – dark cycle at a room temperature of 22 ± 2 °C.

The adoption procedures were performed 48–60 h or 120–132 h after parturition. The biological mother was removed, the pups were counted, sexed using anogenital distance, all males thoroughly mixed with the foster mother’s bedding and placed in a clean warm round cup with a diameter of 15 cm for 20 min. The female pups were put back into their mother’s cage. After that, males were placed with adoptive mothers. Thus, males were raised by heterospecific females, along with female pups of the same species as the adoptive mother. Control pups were treated in the same manner but were caged with their biological mother. The final number of pups per litter, after experimental manipulation, ranged from 4 to 6.

The pups were weaned at 30 days of age. They were housed in same-sex sibling groups (no more than 4 mice per cage) and left undisturbed until 60 days of age. They were then isolated in individual cages. A series of olfactory two-choice tests were performed in the adult offspring at 70–85 days of age, and fMRT investigations were performed at 90–110 days of age (Table 1). In each series of experiments, offspring of at least two litters were used. To prevent exposure to female odors, during experiments all subjects were housed and tested in an all-male rooms.

We refer to different groups by abbreviations: *spicilegus*, *musculus*, *wagneri* – fostered by original mother, *spicilegus*_w_ – *spicilegus* fostered by *wagneri*, *spicilegus*_mus_ – *spicilegus* fostered by *musculus*, *wagneri*_sp_ – *wagneri* fostered by *spicilegus*.

### Urine collection

The samples of urine were obtained from estrus female mice two to three days prior to the experiments or on the day of the experiments by placing the females into small mesh cages with 60-mm Petri dishes under the bottom for 2–4 h. The urine was stored frozen and thawed only once, 1 h prior to the beginning of the experiment. At least five urine donors from each species were used in each series. The stage of estrus was identified evaluating cytology of vaginal smears [79]. The estrus was induced by sequential injections of estrogen and progesterone.

### Behavioral two-choice test: Comparison of con- and heterospecific estrus female odors investigation.

#### Behavioral testing

Males were tested for their odor preference in individual glass chambers (30 × 20 × 20 cm) with a mesh lid and sawdust (2-cm layer) and a piece of cotton wool for nest building at the bottom. The males were placed in the chambers 7 days before the start of the testing. The tests were conducted in the same chambers once in 4–6 days under low intensity artificial illumination, always during the dark-phase of the dark–light cycle to cover the active period of animals, from 8:00 p.m. to 4:00 a.m.

A round plastic stand (diameter 130 mm, height 30.5 mm) was placed near one end of the chamber, and two Petri dishes (40 mm in diameter) were placed on the stand at a distance of 30 mm from one another. A square piece of cellophane (10 × 10 mm) with a drop of donor urine (20 μl) was placed into the Petri dishes immediately prior to the beginning of the experiment. The day before testing, to both habituate the subjects to experimental procedure and to obtain baseline behavioral data, males were tested with clean odor Petri dishes. Males were used in experiments no more than three times. The number of tests and paired combinations of the odors of con- and heterospecific estrus females is given in the corresponding table.

We recorded the time of investigation of each odor source using a stopwatch after the male emerged from the nest for two to three approaches to these odor sources for 10 min. The activity of males, directed to the sources of odor, as a rule ceased in 3–5 min. The observers were blind to the condition of test, and two different observers reached at least 90% inter-observer reliability score prior to register time of odor investigation.

#### Data analysis

Statistical significance of test sample preference based on the time investigation of odor sources was estimated by using nonparametric Wilcoxon Signed-Rank Test for paired samples in UNISTAT Statistical Package Version 6.5.04.

### Investigation of neuronal activation of olfactory bulbs (MEMRI)

#### Animal preparation

House mice of three taxa were divided into fourteen groups (Table. 2). One day prior to testing, the mice were placed in clean ventilated cages (350 × 250 × 120 mm, ZOONLAB GmbH, Germany), with dust-free sawdust as bedding. To test the neuronal activation of MOB and AOB in response to odors 10-μl aqueous solution of 10 mM MnCl^2^ (Sigma-Aldrich Co, MO, USA) was rapidly injected into one nostril using a 20-μl micropipette. After that, the mouse was put into its empty clean cage. Subjects were exposed to either clean “saline” air, or estrus female urinary volatile odors. For the odor stimulation groups, each individual was exposed to one urine odor. All odor and saline exposures were pulsed at 1:3-min on:off (1 min on, 3 min off) intervals taking into account the habituation of glomerular responses in the olfactory bulb following odor stimulation [80]. Each odor was exposed 4 times (16 min in total).

**Table 2:** Summary of treatment conditions, odor exposure, and distribution of subjects

Species of test males and rearing conditions	Test samples (exposure of urine of estrus females or saline)	Number of individuals
*wagneri*	*wagneri*	3
*wagneri*	*spicilegus*	4
*wagneri*_sp_	*wagneri*	5
*wagneri*_sp_	*spicilegus*	5
*spicilegus*	*wagneri*	5
*spicilegus*	*spicilegus*	5
*spicilegus*_w_	*wagneri*	5
spicilegus_w_	*spicilegus*	5
*spicilegus*_mus_	*spicilegus*	2
*musculus*	*musculus*	5
*musculus*	*spicilegus*	4
*musculus*	saline	5
*wagneri*	saline	4
*spicilegus*	saline	4

#### Exposure of odors

For odor exposure, an olfactometer of the following design was used: the air pump (Barbus SB-348A) was connected to the closed cage of the tested individual by a silicone hose with a 1-ml plastic nozzle. A piece of filter paper (0.5 cm × 2 cm) was placed inside the plastic nozzle inserted into the drinking hole in the cage cover and 20 μl of urine / saline of the odor stimulus was applied. During the exposure of the odor stimulus, air was passed through the nozzle at a rate of 200 ml / min. To test each mouse, a new nozzle and a new piece of filter paper were used. For each odor stimulus, a new silicone hose was used.

#### MEMRI: Procedure, parameters and data analysis

We used a 11.7 T BioSpec 117/16 USR (Bruker, Germany) MR-scanner for MEMRI study. The mice were immobilized with a gas mixture (4%) of isoflurane (Isofluran, Baxter Healthcare Corp., USA) and air using an anesthesia machine (The Univentor 400 Anaesthesia Unit, Univentor, Malta) 3 min before the experiment. Anesthetized mouse were placed on a heated surface (temperature 30 °C) set in the MR-scanner. Pneumatic sensor for breathing (SA Instruments, Stony Brook, NY, USA) was put under the lower part of animal body.

**Fig. 1:**
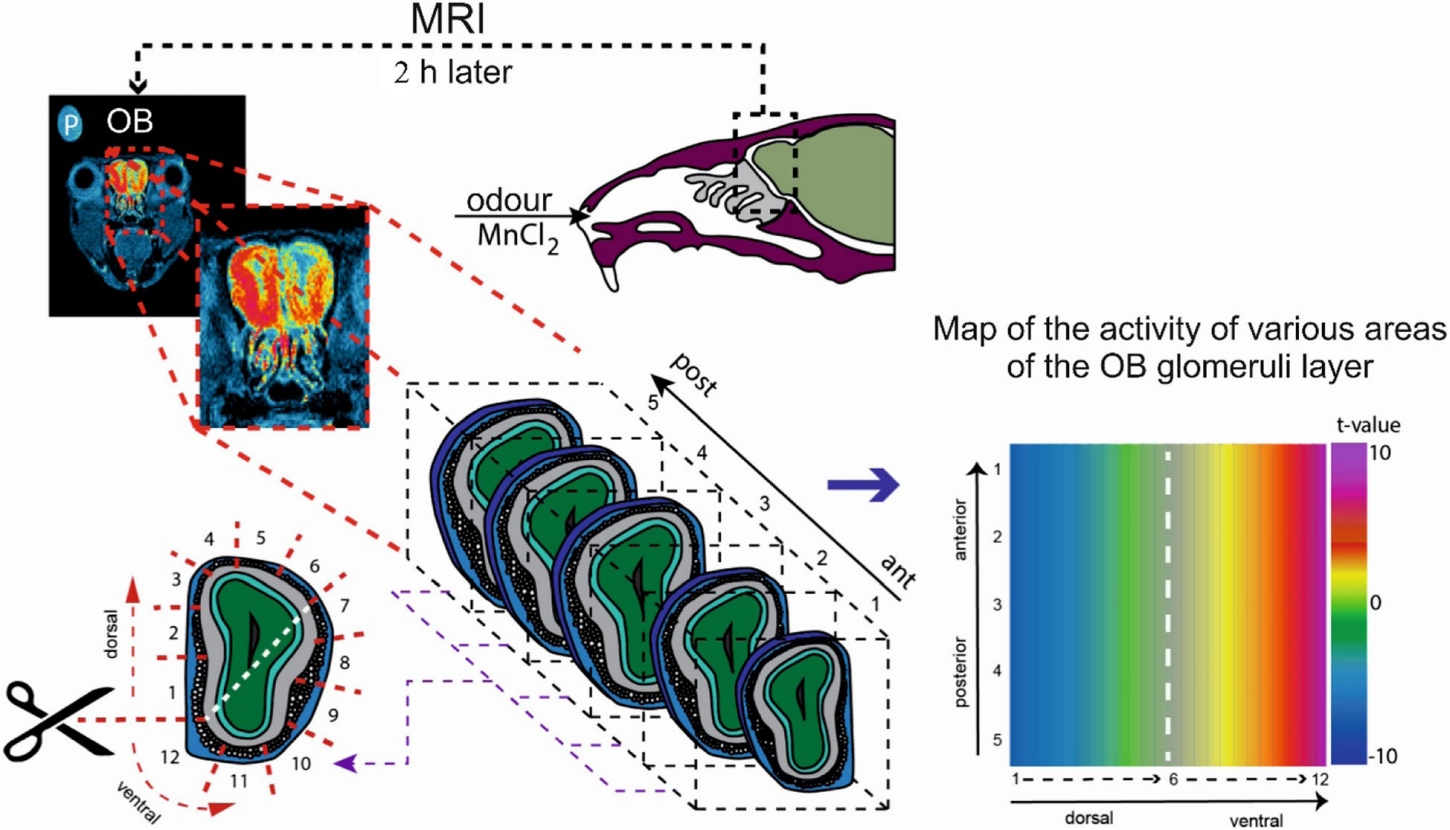
Scanning process of MEMRI and mapping of the activity in the mouse MOB. Maps demonstrate the neuronal activity of various areas of the mouse MOB glomeruli layer in response to the olfactory stimulus

The neuronal activity of olfactory epithelium and VNO of the male *M. spicilegus*, *M. m. wagneri* and *M. m. musculus* was assessed based on the level of the MRI signal in the glomerular layer of the MOB and in the AOB. Accumulation of manganese ions (Mn^2 +^) in neurons of MOB and AOB is very reliably correlated with the level of activity of calcium channels of olfactory epithelial cells and VNO [72, 73]. The accumulation of manganese ions in OB cells was expressed as the ratio of the tissue MRI signal level to the level of the MRI signal in the reference, which was a microtubule with phosphate buffer (0.5 ml) placed along the mouse’s head (Fig. 1). MRI scanning was performed at 2 h after the exposure to the odor stimulus or saline.

The distribution of manganese ions within the OB in control experiments and under the influence of odor stimuli was obtained using T1-weighted images using the RARE (Rapid Acquisition with Relaxation Enhancement) method. Parameters of the pulse sequence of the method (TE = 10 ms, TR = 400 ms), image parameters (field of view – 1.8 × 1.8 cm, matrix – 256 × 256 pixel array, thickness of slice – 0.5 mm, 75 μm × 75 μm × 0.5 mm voxel dimensions, the distance between the slices – 0.5 mm, the number of slices is 5, the orientation of the slices is coronary), the total scan time was 7 min.

Preliminary processing of MRI scans was carried out in ImageJ. This procedure consisted of several stages: aligning the images horizontally, isolating the boundaries of the mouse’s brain, resizing the images. Alignment of the brain geometry made it possible to compare automatically the level of the MRI signal in AOB and in certain regions of the MOB in individuals. To analyze the distribution patterns, the globular layer of OB in each MRI slice was divided into 12 regions. MOB was fitted in 5 scans (Fig. 1). Thus, MOB was presented in 60 regions (12 × 5) and the original resolution of the scan MRI was reduced to 250 μm × 250 μm × 0.5 mm. Within these 60 regions, the MRI level of the signal was averaged. It gave us an opportunity to make various intergroup comparisons and to evaluate the changes in neuronal activity in response to the odor stimulus. Next, a two-dimensional map of the OB was used to visualize the obtained results. The number of the region (1–12) was plotted along the abscissa axis, the cutoff number (1–5) was plotted along the ordinate axis. Pseudo coloring reflects the value of the Student’s t-criterion, characterizing the reliability of the differences between the two groups (Fig. 1). To analyze the activation patterns of the AOB, the following parameters were used: the total number of regions in which the manganese accumulation significantly differs between the two groups, the average value of the t-test and its variance.

To compare the two patterns of the MOB reaction according to the number of regions of the glomerular layer, where the manganese accumulation significantly differs between the two groups (*p* < 0.05, based on the values of the t-test), the χ2 criterion was used. For the values whose variational series approached the normal one, dispersion analysis was used. For multiple average comparisons, the LSD test (Least Significant Difference) was used. Data were expressed as Mean ± SE.

To evaluate the relationship between the two activation patterns of the OB, Spearman’s nonparametric correlation coefficient was used. To compare the two correlation coefficients, the approach described by Myers and Sirois [81] was used.

## Results

### Behavioral testing

Table 3 shows the time that cross-fostered and non-fostered males spent investigating urine samples of con- and heterospecific estrus females during two-choice tests. *M. spicilegus* raised by female *M. m. musculus* spent significantly more time investigating the odor sources of heterospecific females. *Wagneri*-nursed *spicilegus* and *spicilegus*-nursed *wagneri* did not demonstrate significant choice of con- or heterospecific female odor (Table 3). Non-fostered male *spicilegus* investigated longer the odor of conspecific females in comparison with *wagneri-*nursed males (Table 4). *Wagneri*-nursed *spicilegus* investigated longer the urine odor of female *wagneri* in comparison with non-fostered male *spicilegus*. There were no significant differences in time investigation of *wagneri* female odors in *wagneri*-nursed *spicilegus* in comparison with non-fostered male *wagneri,* but time investigation of *spicilegus* female odors by *wagneri*_sp_ were significantly longer than in non-fostered *spicilegus* (Table 4).

**Table 3:** Time of investigation of urine of estrus females by the males

Recipients	Test samples of the urine odor	Time of the odor investigation (average, min – max)	Number of tests		Wilcoxon Signed Rank Test	
			with longer investigation of conspecific odor	total	*P*	Test statistic (*Z*)
*spicilegus*	*spicilegus* *wagneri*	15.7; 0.8–2.8 1.9; 0.3–6.1	13	15	0.0003	−3.2374
*spicilegus*_*mus*_	*spicilegus* *musculus*	7.1; 1.8–15.9 9.7; 3.6–15.1	2	12	0.021	−2.2749
*spicilegus*_*w*_	*spicilegus* *wagneri*	7.8; 2.3–12.2 7.9; 3.9–16.0	11	16	0.865	−0.196
*wagneri*	*wagneri* *spicilegus*	9.1; 1.3–3.1 3.3; 0.9–7.4	11	13	0.0081	−2.5508
*wagneri*_*sp*_	*wagneri* *spicilegus*	8.6; 0.9–21.2 6.8; 2.9–11.2	4	7	0.578	−0.676
*musculus*	*musculus* *spicilegus*	18.1; 1.3–36.1 3.6; 0.3–12.5	15	15	0.0001	−3.4078

See Table 1. Significant values are underlined

**Table 4:** Comparison of the time investigation of odors of con- and heterospecific estrus females by non-fostered and fostered males

Recipients of the odors	Donors of the odors	Time of the odor investigation (average, min – max)	Mann-Whitney U Test	
			*P*	Test statistic (*U*)
*spicilegus* – *spicilegus*_*w*_	*spicilegus*	15.7; 0.8–32.8 7.8; 2.3–12.2	0.0075	62
*wagneri* – *wagneri*_*sp*_	*wagneri*	9.1; 1.3–23.1 8.6; 0.9–21.2	0.8773	43
*spicilegus* – *spicilegus*_*w*_	*wagneri*	2.0; 0.3–6.1 7.9; 3.8–16	0.0000	9
*wagneri* – *wagneri*_*sp*_	*spicilegus*	3.3; 0.9–7.4 6.8; 2.9–11.2	0.0186	16

See Table 1. Significant values are underlined

### Neuronal responses in the MOB by MEMRI

**Fig. 2:**
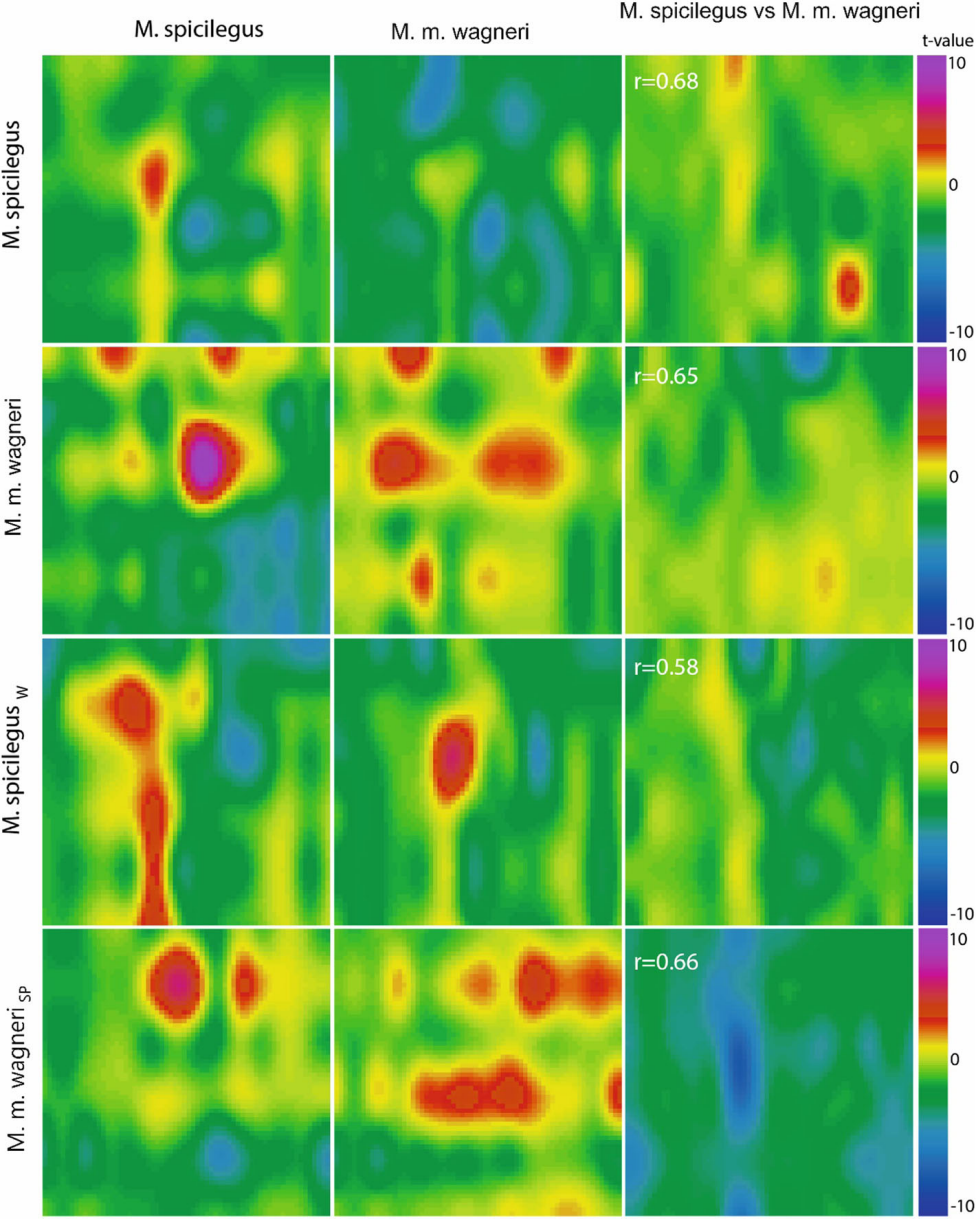
The neuronal responses of *M. spicilegus* and *M. m. wagneri* males to female odors. The patterns of accumulation of manganese ions in areas of the MOB of *M. spicilegus* and *M. m. wagneri* males fostered by con- or heterospecific females in response to the exposure of the urine odor of estrus female *M. spicilegus* and *M. m. wagneri*. The values of the correlation coefficient of the reaction patterns to the odor of *M. spicilegus* and *M. m. wagneri* for each group are indicated. For Figs. 2-5 pseudocoloring illustrates the significance of the increase (*t* > 0, where *t* is Student’s t criterion in pixel-to-pixel comparison of the group mean values of the signal in mouse OB) or decrease (t < 0) of the intensity of contrast accumulation in various MOB areas in response to the olfactory stimulus in comparison with the control group (saline exposure).

**Fig. 3:**
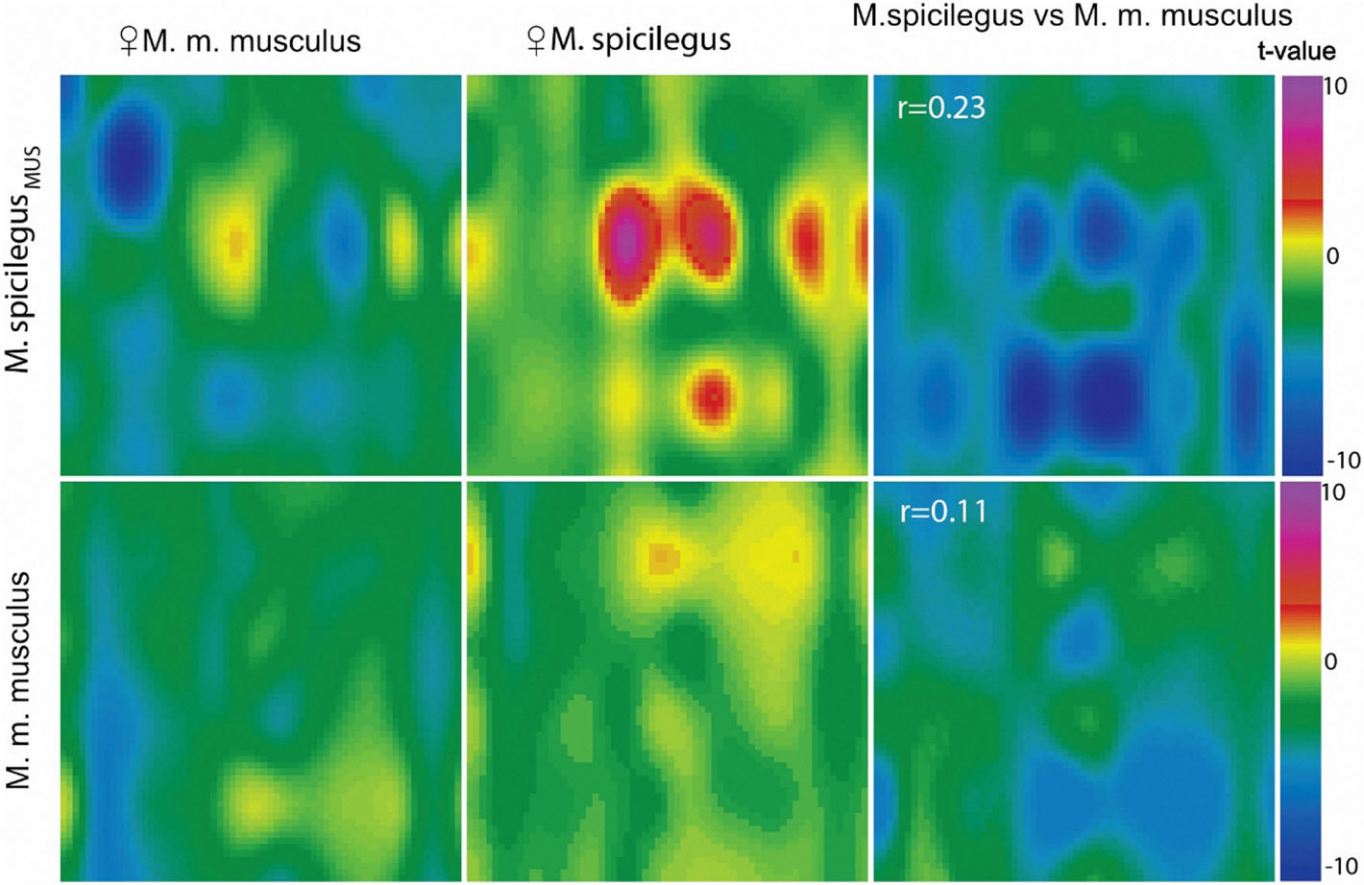
The neuronal responses of *M. spicilegus* and *M. m. musculus* males to female odors. The patterns of accumulation of manganese ions in areas of the MOB in male *M. spicilegus* fostered by *M. m. musculus* (mus - *M. m. musculus*) females and male *M. m. musculus* in response to the exposure of the urine odor of *M. spicilegus* and *M. m. musculus* estrus female. The values of the correlation coefficient of the reaction patterns to the odor of *M. spicilegus* and *M. m. musculus* for each group are indicated

**Fig. 4:**
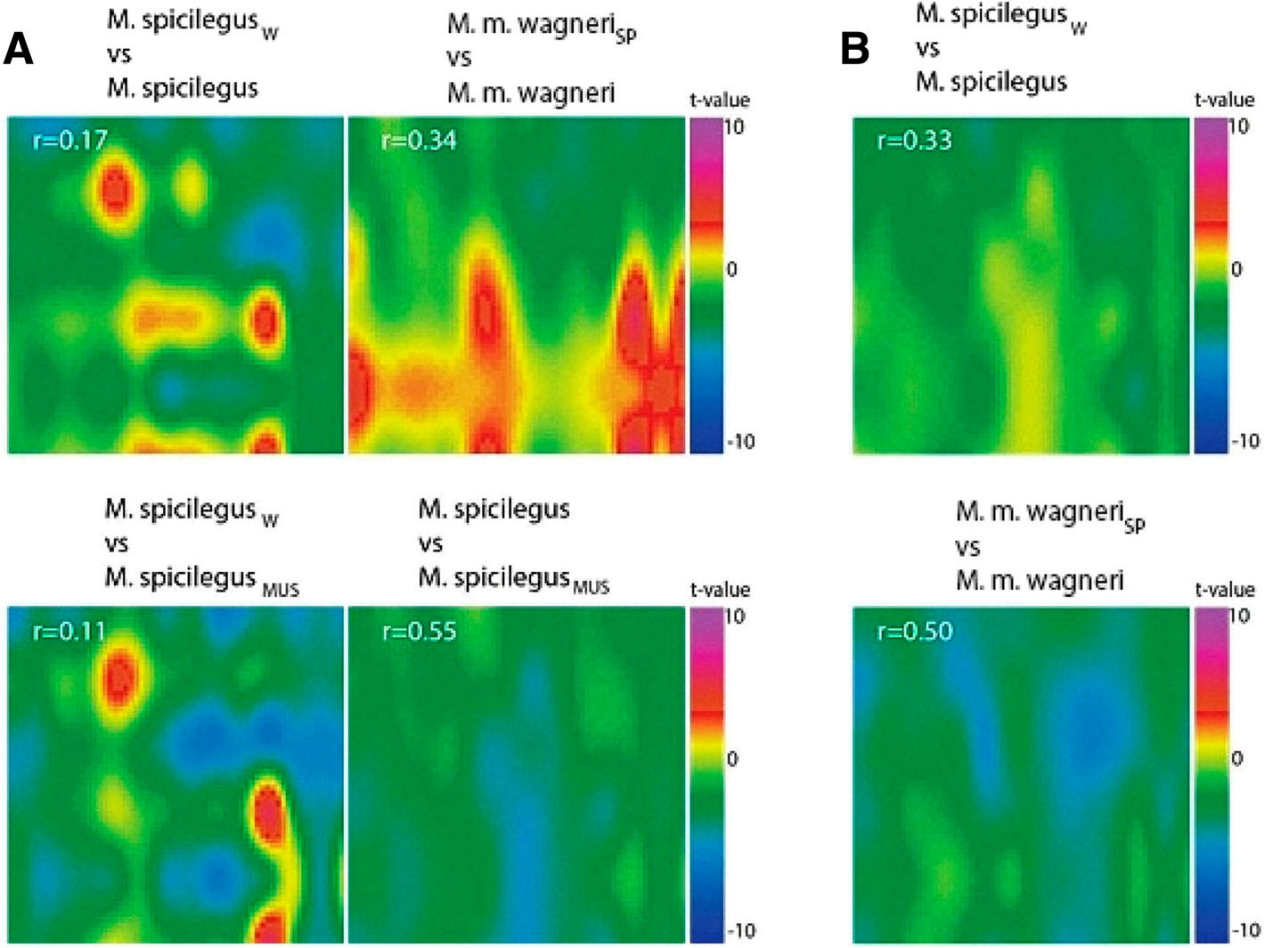
The comparison of the neuronal responses of *M. spicilegus* and *M. m. wagneri* males. The comparison of the patterns of accumulation of manganese ions in areas of the MOB in male *M. spicilegus* (**a**) and male *M. m. wagneri* (**b**), fostered by con- and heterospecific females (sp - *M. spicilegus*, w *- M. m. wagneri*, mus – *M. m. musculus*) in response to the exposure of the urine odor of *M. spicilegus* (**a**) and *M. m. wagneri* (**b**) females. The values of the correlation coefficient of the reaction patterns to the odor of *M. spicilegus and M.m. wagneri* for each group are indicated

“Heat maps” are used to visualize the distribution of the t-test values over the surface of the MOB (Figs. 2 and 3). Table 5 shows the statistical values to compare of the manganese accumulation patterns in MOB in response to the different olfactory cues. All odors induced significant changes in the level of the MRI signal compared to the control (Figs. 2, 3 and 4, Table 5). The number of MOB parts with significant differences of Mn^2+^ accumulation (further in the text «STZ») in male *M. spicilegus* was similar in response to exposure of estrus female urine of *M. spicilegus*, *M. m. wagneri* and saline (χ2 = 1.57, *p* = 0.21). There were no significant differences in the number of STZ in MOB in male *M. m. wagneri* in response to exposure of estrus female urine of *M. spicilegus* and *M. m. wagneri* (χ2 = 1.57, p = 0.21) and in male *M. m. musculus* in response to exposure of estrus female urine of *M. spicilegus* and *M. m. musculus* (χ2 = 2.31, *p* = 0.12).

**Table 5:** Patterns of accumulation of the manganese in OB of the males in response to the odor urine of females

Odor vs control	t-test mean value	Standard error of the mean	Variance	STZ
M. m. musculus*				
*spicilegus*	0.52	0.10	0.61	9
*musculus*	−0.33	0.10	0.69	3
M. spicilegus*_mus_				
*musculus*	−0.88	0.17	1.94	10
*spicilegus*	1.02	0.15	1.44	9
M. spicilegus*_w_				
*spicilegus*	0.50	0.16	1.61	13
*wagneri*	0.29	0.12	0.97	3
M. spicilegus*				
*spicilegus*	0.52	0.10	0.61	4
*wagneri*	−0.31	0.09	0.55	3
M. m. wagneri*_sp_				
*spicilegus*	0.76	0.14	1.22	7
*wagneri*	1.45	0.13	1.18	22
M. m. wagneri*				
*spicilegus*	1.50	0.12	0.89	19
*wagneri*	0.70	0.19	2.30	12

*STZ* suprathreshold zones – zones of OB, in which MRI signal level significantly differ from those in the control (Student’s t-test, *p* < 0.05), * – recipients of the odors

The intensity of the response (expressed as the number STZ in MOB) is significantly higher in male *M. m. wagneri* compared to *M. spicilegus* as to odor of the urine of *M. spicilegus* females, and to the odor of female *M. m. wagneri* (χ2 = 3.77, *p* = 0.05).

Number of STZ in male *M. spicilegus* fostered by heterospecific females increased in comparison with a male *M. spicilegus* nursed by their mother in response to urine of estrus conspecific females. This increase was significant in the male *M. spicilegus*_w_ (Table 5, χ2 = 4.39, *p* = 0.03) and did not achieve significant differences in the male *M. spicilegus*_mus_ (χ2 = 2.02, p = 0.1). Number of STZ in these groups of males did not change in response to exposure of female odor of *M. m. wagneri* (Table 5). In male *M. m. wagneri* fostered by female *M. spicilegus* number of STZ increased significantly in response to the odor of conspecific females in comparison with male nursed by their mother (χ^2^ = 4.07, р = 0.04) and decreased to exposure of urine of female *M. spicilegus* ((χ^2^ = 5.94, р = 0.01).

**Fig. 5:**
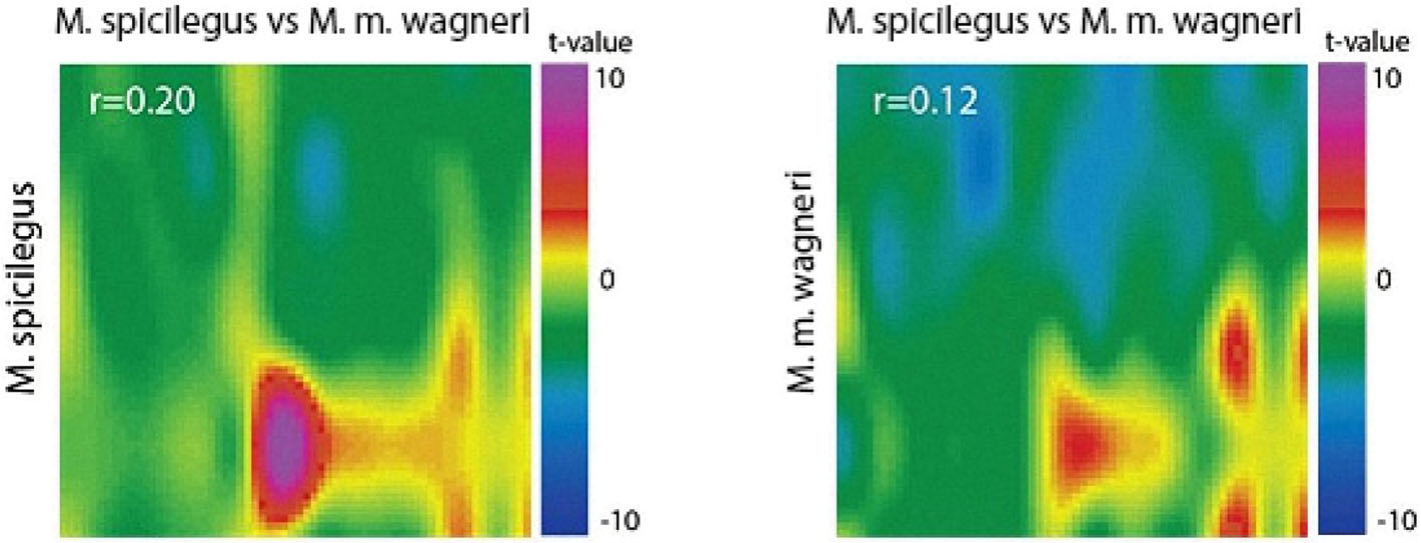
The interspecific comparisons of the neuronal responses of *M. spicilegus* and *M. m. wagneri* males. The interspecific comparisons of the patterns of accumulation of manganese ions in various areas of the MOB in male *M. spicilegus* and male *M. m. wagneri* in response to the exposure of the urine odor of *M. spicilegus* and *M. m. wagneri* estrus female. The values of the correlation coefficient of the reaction patterns to the odor of *M. spicilegus* and *M. m. wagneri* for each group are indicated

The patterns of reaction to the same odors between male *M. spicilegus* and male *M. m. wagneri*, nursed by con- and heterospecific females of these species (Figs. 4 and 5) showed significantly lower correlation coefficients compared to responses to different odor stimuli within each group (Fig. 2, Table 6). Correlation analysis showed that there was a strong quantitative similarity between the reaction patterns in male *M. spicilegus* and *M. m. wagneri*, fostered by con- and heterospecific females, elicited by the odors of the estrus urine of con- and heterospecific females (Table 6, Fig. 2).

**Table 6:** Comparison of partial correlation coefficients of the patterns of activation of the OB neurons in mice of different species in response to urine odors of con- and heterospecific females

Species of the test males	Test sample of urine of females	Comparison of experimental groups	Partial correlation coefficients		*P*- value
			*r*_*1*_	*r*_*2*_	
*spicilegus*	*spicilegus*	*spicilegus* *spicilegus*_w_	0.17	0.68	0.03
		*spicilegus* *spicilegus*_mus_	0.55	0.68	0.31
		*spicilegus*_*mus*_ *spicilegus*_w_	0.11	0.68	0.02
	*wagneri*	*spicilegus* *spicilegus*_w_	0.33	0.68	0.09
*wagneri*	*wagneri*	*wagneri* *wagneri*_sp_	0.34	0.65	0.12
	*spicilegus*	*wagneri* *wagneri*_sp_	0.50	0.65	0.3

### Neuronal responses in AOB by MEMRI

**Fig. 6:**
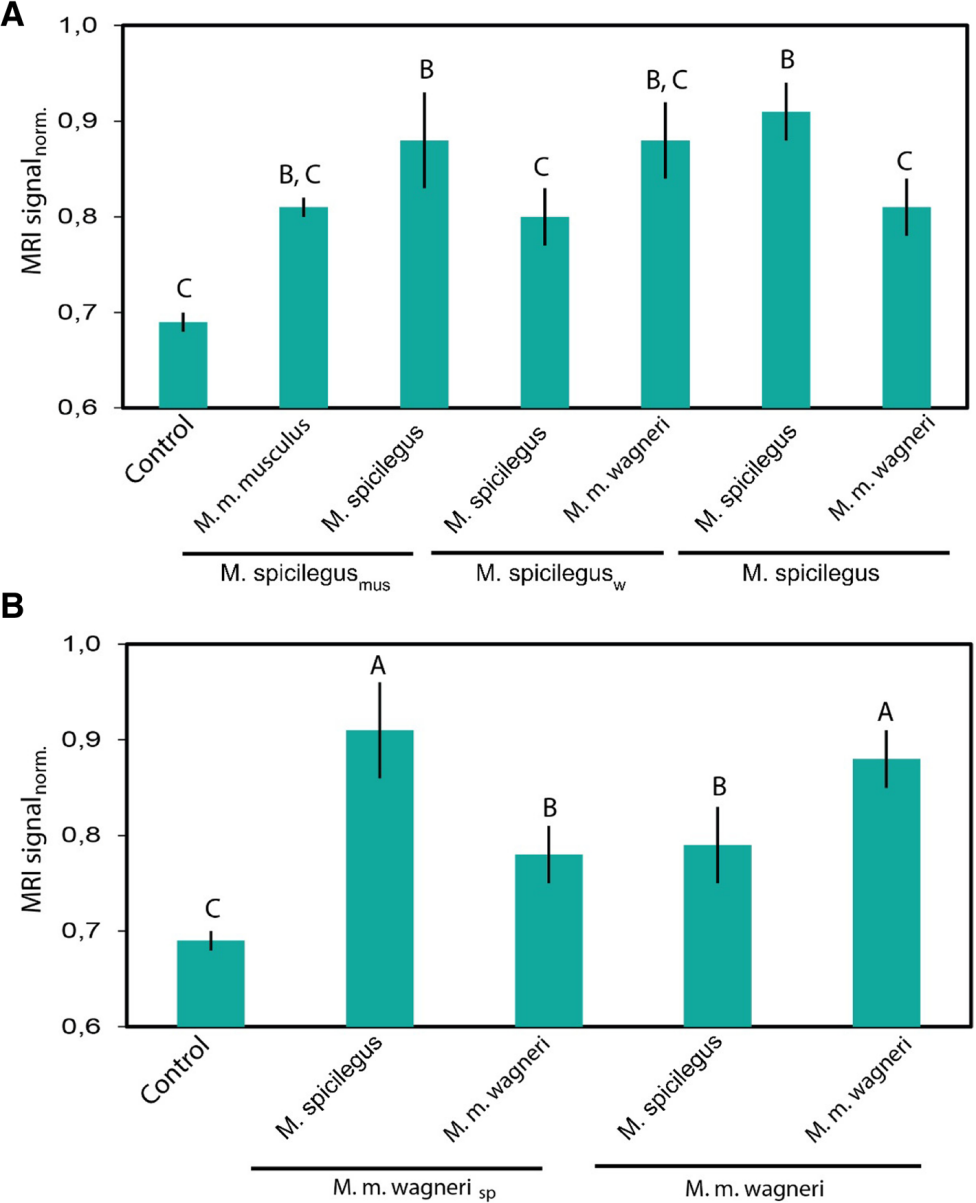
The neuronal activation of the AOB of males. The neuronal activation of the AOB of males of *M. spicilegus* (A) and males of *M. m. wagneri* (B), fostered by con- and heterospecific females (sp - *M. spicilegus*, w - *M. m. wagneri*, mus - *M. m. musculus*), in response to the odor of urine of estrus females. As a criterion for estimating the response of neurons to the odor stimulus, the intensity of accumulation of manganese ions was used, which is proportional to the level of the MRI signal in this region. A, B, C - significant differences in the LSD test (*p* < 0.05)

**Fig. 7:**
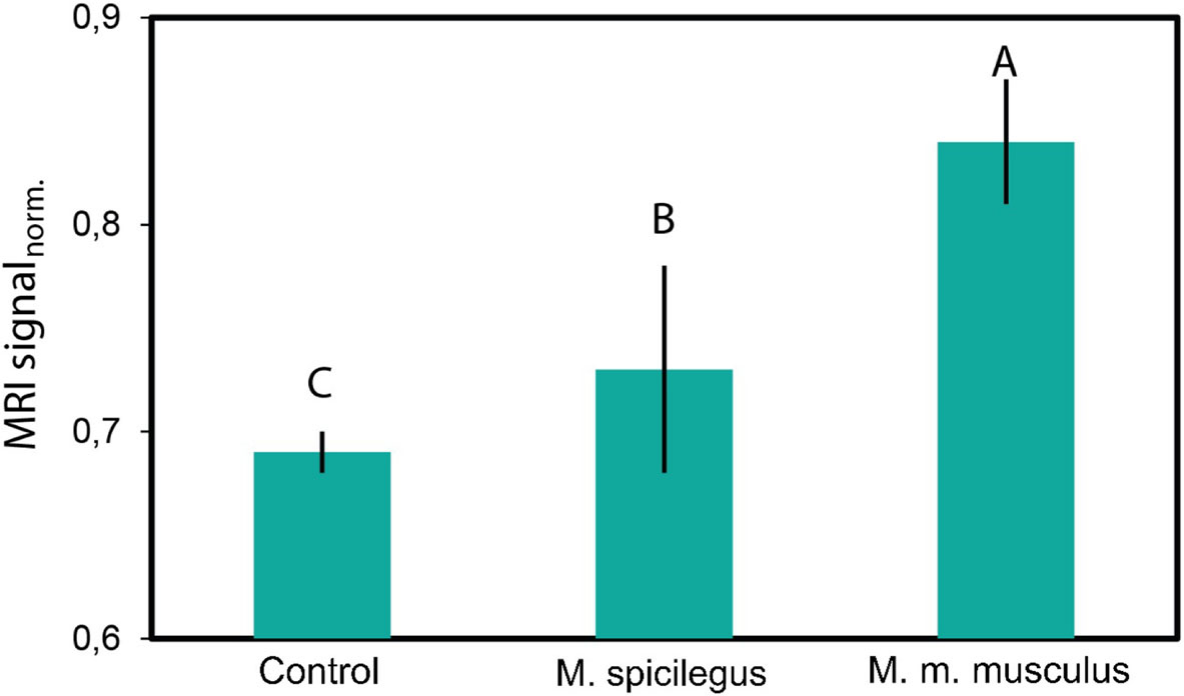
Neuronal activation of the AOB of male *M. m. musculus.* The response to the odor of urine of estrus females. As a criterion for estimating the response of neurons to the odor stimulus, the intensity of accumulation of manganese ions was used, which is proportional to the level of the MRI signal in this region. A, B, C - significant differences in the LSD test (*p* < 0.05)

The level of MRI signal, obtained from the evaluation of manganese accumulation in AOB neurons, was significantly higher for non-fostered males when exposed to the urine odor of conspecific estrus female than when exposed to heterospecific females (Figs. 6 and 7). The response pattern was the opposite in males fostered by heterospecific females.

## Discussion

The change of the response pattern of AOB neurons of males fostered by heterospecific females to the urine odor of con- and heterospecific females, on the opposite is one of the most impressive results of our studies. We demonstrated that the maternal environment, including odor, has a greater effect on the MRI level of the signal in the AOB than the genetic relationship of the recipient and the donor of the odor stimulus.

The vomeronasal or accessory olfactory system has long been considered to be tuned for sensing pheromones and indeed all chemically identified house mouse pheromones are detected by V1R and V2R receptors of VNO [82–84], see reviews [28, 58, 85]. Urine, a well-characterized pheromone source in mammals, as well as saliva, activates AOB neurons in a manner that reliably encodes the donor animal’s sexual and genetic status [86]. It has been already accepted earlier that the two chemosensory systems, the main olfactory and the vomeronasal system, were responsible for different functions. The main olfactory system was considered to be responsible for recognizing the volatile odorant molecules, used in the context of social communication. Current views on olfactory processing suggest that both the main olfactory and vomeronasal systems detect partially overlapping sets of social chemosignals. Consequently, both systems should be conceived as complementary rather than as separate pathways [87, 88]. The further studies have demonstrated that both chemosensory systems are involved in pheromone detection [88–91]. The exposure of some pheromones induced activation in the glomerular layer of MOB in house mice and rats [92, 93].

We have also shown that the changes in neuronal activation under the influence of the early olfactory experience in response to the urine odor of estrus con- and heterospecific females occur in the MOB.

Induced sensitivity to olfactory stimuli in mammals has been proved experimentally both for different substances (odorants and pheromones) and for complex mixtures, such as animal excreta [94, 95]. This phenomenon is associated the plasticity of the processes of chemical communication of mammals. The induced sensitivity to olfactory stimuli is highly specific and does not affect the overall olfactory sensitivity and sensitivity to substances structurally or functionally unrelated to the exposed substances [94]. The exposure of individual urine samples within the two-week timeframe after eyes open increased sensitivity to target samples by 100-fold relative to controls (no exposure), while similar exposures to individual urine samples during adulthood or first 10–14 days of life increased sensitivity to target individual urine samples by 10-fold only [94, 95]. The timing of sensitive period for maximal imprinting of conspecific odor is also confirmed in Norway rats *Rattus norvegicus* [96]. Sensitization to individual odors in mice also was demonstrated for heterospecific urine samples. Mice cross fostered by Djungarian hamsters (*Phodopus sungorus)* from the birth to the time of weaning, were able to discriminate individual hamster urine sample at the concentration of 3–4% which is very close to discrimination thresholds for conspecific urine in mice (2–3%) [94]. A number of studies demonstrated the involvement of both peripheral and central mechanisms into processes of sensitization to the odors [97–100]. According to these data sensitive period toward individual odors begins in pups after two weeks of early postnatal development. Moreover, the time limits of the critical period correspond to the period of maturation of the synaptic apparatus of the olfactory system [101]. During the period from the 11th to the 20th day of development that a sharp increase in the number of dendrites in the olfactory system occurs [102]. In the mouse OB starting from the 14th day after birth mitral cells reorient their cell bodies which followed by growing of the first definitive dendrites from their soma and forming of the first synapses. Day later considerably greater numbers of axo-dendritic and a few dendrodendritic synapses occur in the presumptive glomerular layer [102]. It has been shown for several species of mammals, that the maturation of synaptic contacts in the olfactory system correlates to behavioral patterns of imprinting [103, 104]. Long exposures (1 week or more) of pure androstenone (male boar pheromone) cause long-term changes in the olfactory sensitivity of house mice at the level of behavior and irreversible changes at the level of the olfactory lining [105, 106]. Modern studies have identified possible neuroanatomical correlates of plasticity in the olfactory analyzer [107]. Methods of molecular biology demonstrated the possibility of switching the expression of one olfactory receptor gene to another in one sensory neuron in a certain sensitive period [108]. Our data are in good agreement with these findings. The difference in the reaction of cross-fostered mice in our experiments may be due to changes at the receptor level or the formation of new pathways of the accessory and main chemosensory systems during the sensitive periods under the influence of the early olfactory experience.

It is more difficult to explain elevated number of STZ in male *M. spicilegus* and *M. m. wagneri* fostered by heterospecific females compared to a males nursed by their mothers in response to urine of estrus conspecific females.

Urine is a very complicated mixture consisting of hundreds of substances, including pheromones and odorants. Currently, there is not enough information about the mechanisms of perception and encoding of complex mixtures in main and accessory olfactory systems. The problems are at relatively early stages of the study [109]. For example, in mice individual neurons in AOB activated selectively by specific combinations of the sex and strain of conspecifics [110]. Authors infer that mammals encode social and reproductive information by integrating vomeronasal sensory activity specific to sex and genetic makeup. Much less is known about these processes under the influence of the early olfactory experience. It was shown that early experience with multiple odorants results in increased responsiveness both to previously experienced odorants and to novel odorants that stimulate previously activated regions of the bulb [111].

Chemical composition of urine of *M. domesticus* and *M. spicilegus* has qualitative and quantitative differences. A series of volatile and odoriferous lactones and the presence of coumarin were the unique features of *M. spicilegus*, as was the notable absence of 2-s-butyl-4,5-dihydrothiazole (a prominent *M. domesticus* male pheromone) and other sulfur containing compounds. Some other *M. domesticus* pheromone components were also found in *M. spicilegus* urine but in other concentrations [59].

It is possible that increase of number of STZ in male *M. spicilegus* and *M. m. wagneri* fostered by heterospecific females in comparison with a males nursed by their mothers in response to urine of estrus conspecific females was due to odor novelty. Exposed odor of conspecific estrus female should be novel for cross-fostered males.

The change of the behavioral preference for con- and heterospecific female odors of males fostered by heterospecific females is consistent with the results on neuronal activation in OB and with our previous data. We have demonstrated that preference of the odor of the potential mate partner has changed in cross-fostered *M. musculus* and *M. spicilegus* [37]. In these studies, a shift in preference toward odor of the foster species was due to increased attraction to the heterospecific foster species and decreased attraction to the conspecific species in some cases (Table 4).

The differences of olfactory signals in closely related taxa at early stages of divergence may be the first step of the development of their reproductive isolation. The process of reinforcement is one of mechanisms of speciation in which learning can play an important role [6, 112]. When two populations have accumulated differences in allopatry, but come into secondary contact before the speciation process is completed, mated pairs that hybridize between them and their descendants may have reduced fitness. Reinforcement occurs when this lowered fitness of hybridizing pairs and hybrids drives the evolution of premating isolation to prevent hybridization [113–116]. Reinforcement may be affected by learning in a number of ways (for reviews see Servedio et al. [117]). For example, paternal imprinting [118] can drive speciation and maintain species differences easily compared to genetically inherited preferences, even sex-linked ones. According to the model of Servedio et al. [117], reinforcement can indeed occur via imprinted preferences.

We altered the maternal environment by cross-fostering and demonstrated that behavioral and neuronal responses to con- and heterospecific odors changed in closely related taxa of Mus as result of early experience. We demonstrated the importance of early learning in the mate preference for odor of mice in adulthood and the possibility of epigenetic contribution in the formation of precopulatory isolation. In our experiments we used relatively strongly divergent and non-interbreeding in nature species. Nevertheless, a change in the preference of the odor of the mate, and probably the mate choice in *M. musculus* under the influence of early experience, could be realized in the case of secondary contact with *M. domesticus.*

These data allow us to suggest the following scenario of development of precopulatory isolation in evolution of house mice. According to the hypothesized history and differentiation of *M. musculus* and *M. domesticus*, initial colonization of the Middle East from the Indian cradle was followed by the phase of isolation and subsequent divergence. The phases of isolation and secondary contact alternated in the process of evolution. Secondary contact and gene flow occurred during interglacial periods and isolation occurred during glacial periods [119]. Allopatric populations could have a divergence of odors.

During secondary contact of isolated before populations (in interglacial periods) individuals could prefer mate partner with imprinted odor. Gradually, preference for such altered odor could be fixed in the course of evolution. According to one of the models when reinforcement occurs via the evolution of population specific traits, if imprinting is already established, greater trait differences between populations would be expected when there is lower hybrid fitness [117]. If hybrids had reduced fitness, the process of reinforcement could drive natural selection against hybrids. Indeed, the effect of reinforcement selection was shown in the European hybrid zone of *M. musculus* and *M. domesticus* [120].

According to current studies, individuals of *M. musculus* populations from the border of the European hybrid zone consistently show assortative sexual preference of conspecifics, as well as mice from the central parts of the range [48, 121, 122]. The pattern is more variable in *M. domesticus*, with some populations showing assortative preference [121] and others do not demonstrate it [46, 48, 123].

On the other hand, early olfactory experience in hybrid zones should favor mate choice of hybrids. This preference may affect the relative stability of such zones. The patterns of preference differ between natural hybrids from Denmark and F_1_ hybrids obtained in the laboratory from mice of the same geographical origin. The natural hybrids demonstrate *domesticus*-like preference [121], while the two reciprocal F_1_ crosses do not show a consistent preference, but when a preference is detected it is *musculus*-like [46]. These differences can indicate the possibility of significant differences at the first stage of secondary contact of populations, when the majority of hybrids are F_1_ and backcrosses, as compared to today’s highly recombined genotypes [124].

The evolutionary history of *M. spicilegus* is poorly understood. At present, it is not possible to reconstruct the pattern of interactions of the ancestral forms of free-living species group of mice, which includes *M. spicilegus*, and the synanthropic species group including *M. musculus* and *M. domesticus*. Researchers disagree on the origin and migration routes of ancestral forms of free-living species [125–128]. Apparently, the ancestral forms of these two subsequently divergent groups (synanthropic and free-living species) could inhabit the same territories, and that does not exclude the influence of the role of learning at an early age on the formation of mechanisms of precopulating isolation between species.

Thus, early learning could play a role in the formation of mechanisms of precopulatory reproductive isolation between closely related taxa of house mice in the *Mus musculus* s.l. species group in the process of evolution.

## Conclusions

The change in the maternal environment alters the reaction patterns to con- and heterospecific odors in adult specimens of *M. spicilegus* and *M. musculus*. Non-cross-fostered males of three taxa demonstrated a strong preference for odor of conspecific females compared to odor of heterospecific ones. *Spicilegus*-nursed *musculus* preferred odor of heterospecific females. *Wagneri*-nursed *spicilegus* and *spicilegus*-nursed *wagneri* did not demonstrate significant choice of con - or heterospecific female odor.

The level of MRI signal, obtained from the evaluation of manganese accumulation AOB neurons, was significantly higher for non-fostered males of *M. spicilegus* and *M. m. wagneri* when exposed to the urine odor of conspecific estrus female than when exposed to the urine odor of heterospecific female.

The response pattern of neuronal activation in AOB of males fostered by heterospecific females to the urine odor of con- and heterospecific females, was the opposite of the response pattern of non-fostered males. The maternal environment, including odor, has a greater effect on the MRI level of the signal in the AOB of adult males than the genetic relationship of the recipient and the donor of the odor stimulus.

The neuronal activation in the MOB in males of *M. spicilegus* and *M. m. wagneri* in response to the odor of con- and heterospecific estrus females is significantly changed by the maternal environment (cross-fostering) during early postnatal ontogenesis.

The early learning coming from the maternal environment could play a role in the formation of mechanisms of precopulatory reproductive isolation between closely related taxa of house mice in the *Mus musculus* s.l. species group in the process of their evolution.

## Abbreviations

AOB: Accessory olfactory bulb
F_1_: Hybrids of the first generation
fMRI: Functional magnetic resonance imaging
MEMRI: Manganese-enhanced MRI
MOB: Main olfactory bulb
OB: Olfactory bulb
*spicilegus*_mus_: *Spicilegus* fostered by *musculus*
*spicilegus*_w_: *Spicilegus* fostered by *wagneri*
STZ: The number of MOB parts with significant differences of Mn^2+^ accumulation
*wagneri*_sp_: *Wagneri* fostered by *spicilegus*
